# Oat-Based Foods: Chemical Constituents, Glycemic Index, and the Effect of Processing

**DOI:** 10.3390/foods10061304

**Published:** 2021-06-07

**Authors:** Kailong Zhang, Rui Dong, Xinzhong Hu, Changzhong Ren, Yuwei Li

**Affiliations:** 1 Department of Food Engineering and Nutrition Science, Shaanxi Normal University, Xi’an 710119, China; zkailong95@163.com (K.Z.); rell_dr@126.com (R.D.); 2 Baicheng Academy of Agricultural Sciences, Baicheng 137000, China; 13500830080@163.com; 3 Guilin Seamild Food Co., Ltd., Guilin 541000, China; liyuwei@seamild.com.cn

**Keywords:** oats, functional food, glycemic index, β-glucans, processing

## Abstract

The desire for foods with lower glycemic indices has led to the exploration of functional ingredients and novel food processing techniques. The glycemic index (GI) is a well-recognized tool to assess the capacity of foods to raise blood glucose levels. Among cereal crops, oats have shown the greatest promise for mitigating glycemic response. This review evaluated decades of research on the effects of oat components on the GI level of oat-based foods with specific emphasis on oat starch, β-glucans, proteins, and phenolics. The effects of commonly used processing techniques in oats on GI level, including heating, cooling, and germination were also discussed. In addition, the GI of oat-based foods in various physical formats such as whole grain, flakes, and flour was systematically summarized. The aim of this review was to synthesize knowledge of the field and to provide a deeper understanding of how the chemical composition and processing of oats affect GI, thereby further benefiting the development of low-GI oat foods.

## 1. Introduction

**Figure 1:**
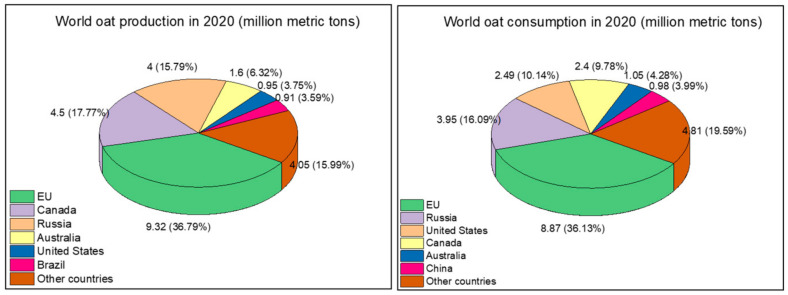
World production and consumption of oats.

Oats have earned a worldwide reputation as a healthy and nutritious food and as a valuable livestock feed due to its high protein content. The total global production of oats ranks sixth among grain crops, following that of corn, wheat, rice, barley, and sorghum. Although most oat production is used as livestock feed, oats are suitable for human consumption and have many applications, including as oatmeal and oat flour. The crop is mainly cultivated in temperate regions of the northern hemisphere. Most cultivated oats are varieties with the seed covered by a hull (*Avena sativa*), whereas varieties cultivated in China are mainly without hulls (*Avena nuda*), which is often called naked oat [1]. In 2020, the global production of oats was 25.33 million metric tons with 10.65% of growth from 2019 to 2020. The EU is the largest oat producer, followed by Canada, Russia, Australia, the United States, and Brazil. The largest oat consumers are the EU, Russia, the United States, Canada, Australia, and China. In the past five years, annual production and consumption of oats reached a steady state of more than 20 million metric tons (Figure 1) [2].

Oats contain about 60% starch, 14% protein, 7% lipids, and 4% β-glucan. In contrast to other grain crops, oats are high in protein and lipids. The distinguishing feature of oats is its rich content of dietary fiber, especially soluble β-glucans. As for the micronutrients, oats are particularly rich in potassium. Other minor components such as phenolics are also present in oats. Among them, avenanthramides, which are unique to oats, have attracted enormous interest [1]. For decades, many scientific studies have established the health benefits of oats in lowering glycemic response, reducing blood cholesterol level, balancing gut microbiota, and regulating blood pressure. In 1997, a landmark health claim was approved by the Food and Drug Administration (FDA) in the United States indicating that soluble fiber from oat products can help reduce the risk of heart disease. Among this scientific research, special emphasis has been given to the beneficial effects of oats on blood glucose levels. As expected for a cereal grain food, oat grains are rich in carbohydrate with starch content ranging from 51% to 65% [3]. Oats are consumed most commonly as whole grains where nutrients are well retained, and their consumption is recommended for the prevention of diabetes. In some regions, such as Inner Mongolia in China, oats are consumed as a staple food.

Diabetes is a chronic metabolic disease and has become a threat to human health globally. The number of people with diabetes has increased greatly in the past three decades. It has been reported that 422 million people worldwide suffer from diabetes, and each year, 1.6 million deaths are directly associated with the disease. Type 2 diabetes, generally resulting from resistance to insulin, is the most common form of the disease [4]. Controlling blood glucose is one of the most crucial means of prevention and treatment of Type 2 diabetes, and diet is a primary tool for managing blood glucose. Consuming foods that do not quickly raise blood glucose can help prevent the occurrence of diabetes. In contrast, the risk of developing diabetes increases with the consumption of food that is quickly metabolized to produce glucose in the blood. To evaluate the capacity of food to affect blood glucose, the concept of the glycemic index (GI) was first proposed by Jenkins et al. [5]. GI describes the effect of consuming a given food on blood glucose level compared to that induced by pure glucose. Foods with GI values of less than 55 are considered low GI, 56 to 69 are mid-range GI, and those with GI values greater than 70 are defined as high GI foods. GI is a rating tool for the glycemic rising capacity of a food and it provides a method to identify food useful for the management of Type 2 diabetes [6]. The glycemic load of a food serving can be used to assess the blood glucose rising capacity of each serving of a meal, and it is calculated by multiplying the carbohydrate content of the serving by its GI [7]. Low-GI foods generate a flatter postprandial glucose level with more smooth peaks. The day-to-day consumption of low-GI foods is able to help manage long-term blood glucose levels for patients with Type 2 diabetes, because low-GI foods are effective in controlling fasting blood glucose and glycated hemoglobin A1c [8]. As for diabetes prevention, higher-GI diets increased the risk of developing Type 2 diabetes, whereas the low-GI diets showed a protective effect over this disease, according to a meta-analysis examination [9].

Over the past few decades, the beneficial effects of oat consumption on blood glucose levels have been established by many researchers. GI became an effective tool to assess the relative effects of various oat products. Several researchers have reviewed the GI of foods such as gluten-free bread and products made from starchy crops [10,11]. Oats are habitually regarded as the low-GI ingredients, but the GI value is collectively determined by chemical composition and processing conditions. Previous literature has systematically summarized the GI values of oats with particular emphasis on oat flakes [12]. However, there are many oat products in commercial markets, such as oatmeal, oat rice, and oat milk. These diversified oat products, accompanied by various processing treatments, generally show differences in GI values. Knowledge of the GI of other oat products such as grains, flours, and bran has rarely been evaluated. Moreover, the chemical composition of food fundamentally determines the GI value. A recent review of the GI of starchy foods from the perspective of chemical composition concluded that resistant starch and phenolics contributed greatly to GI [13]. Plenty of plant-derived polysaccharides have been proved to play a significant role in managing glucose and insulin metabolism. For example, arabinoxylan extracted from cereal grains is able to decrease postprandial glucose and insulin response owing to its high viscosity [14]. However, there has been no previous review of how the GI of oat products is influenced by the unique nutritional and chemical composition of oat grain. The effect of processing treatments, such as heat treatment and germination, remains unclear as well. Therefore, the present review systematically summarizes the relation between oat chemical composition, especially oat β-glucans, and GI. The effect of processing on GI, such as food structure, heating, and cooling, is evaluated by considering how ingredients are altered. In addition, the GI of different oat products with various structural formats including grains, flakes, flours, and bran is thoroughly reviewed. We hope our review will contribute to a better understanding of the multi-faceted complexity of the GI of oat-based foods.

## 2. Chemical Constituents of Oats and Their Effects on GI

**Figure 2:**
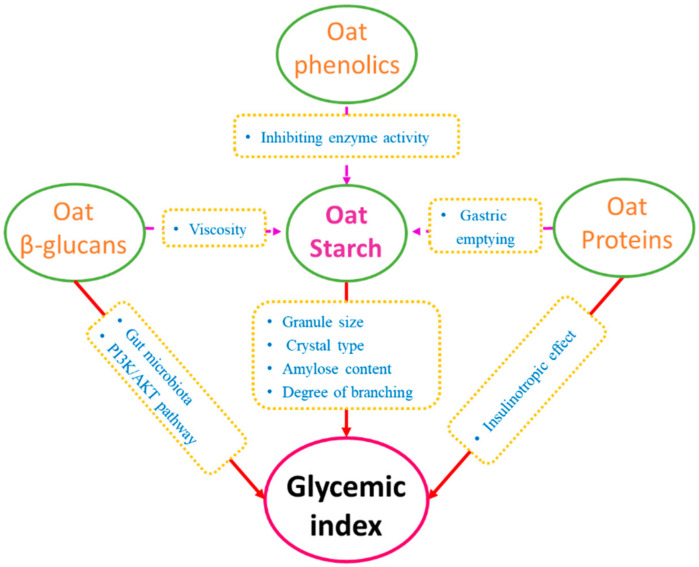
Schematic diagram of representative oat components that can influence GI.

Oats are composed of diverse nutrients, including macronutrients such as proteins, starch, and other polysaccharides, and micronutrients, such as phenolic compounds and vitamins. As a typical grain crop, oats are rich in both available carbohydrates that the human body can absorb and utilize, particularly starch, and unavailable carbohydrates that cannot be digested in the human upper gastrointestinal tract, such as β-glucans [15]. The abundant starch content in oats has a potent potential for releasing glucose in the bloodstream. However, several bioactive components, such as oat β-glucans and phenolics, can prevent the GI from rising via several mechanisms. A more recent study also indicated that consuming oat polar lipids could reduce glucose and insulin responses and modulate second meal postprandial metabolic responses [16]. Multi-faceted factors contributed by oat components affect the GI level of final foods. Figure 2 is a schematic showing the possible influences of several typical oat components on GI level. In this section, the effects of several representative oat components on the GI level are discussed.

### 2.1. Oat Starch

Starch is the major glycemic carbohydrate in the human diet, and its quality and quantity are the largest determinants of a food’s GI level. Many factors, such as crystallinity, the amylose to amylopectin ratio, and molecular weight, determine the digestion rate and the final glycemic behavior of a starch. As in other grains, starch is the most abundant component in oats, accounting for nearly 60% of the total dry weight. Due to the variation generated by plant genotype and production environment, the contents of starch in oats ranges from 51% to 65% [3].

Starch can be classified by its digestion rate as rapidly digestible starch (RDS), slowly digestible starch (SDS), and resistant starch (RS). RDS refers to the starch that releases glucose in the first 20 min of enzymatic hydrolysis. SDS is digested in the small intestine, where most of its glucose is slowly released between 20 and 120 min of enzymatic hydrolysis. In contrast, RS is not digested in small intestine but instead can be utilized in the colon by gut microbiota [17]. The proportion of SDS in natural oat raw starch is 40% [18]. The concept of SDS is method-oriented in that the values are dependent on the method used to measure the starch digestion rate. The mechanism that produces SDS is related to the accessibility of enzymes to the starch macromolecules as affected by the physical and chemical structure of starch.

The physical structure of starch, including its polymorphism and morphology, is dominated by its crystallinity. As processing such as heat treatment can greatly alter the starch crystals, here, we mainly discuss native raw oat starch. Many studies have confirmed that raw oat starch consists of the A-type polymorph [19,20] with a relative crystallinity of 23.3% [19]. Since A-type starch contains more short amylopectin chain units, oat starch should be more susceptible to digestion by enzymes. In addition, oat starch granules are much smaller than those of wheat, barley, and corn, ranging from 3 to 10 μm [21], and these smaller granules should interact with enzymes more easily due to the higher specific surface area. Morphologically, oat starch granules exhibit irregular and oval shapes similar to those of rice starch. These features of oat starch contributed to an increased GI behavior. The estimated GI (eGI) of raw oat starch was 91 according to the report of Kaur et al., which is higher than that of wheat and maize starch [18]. Despite this relatively high GI, the GI value of oat-based foods generally appears low due to the complexity of oat food composition.

Starch digestion behavior is also influenced by chemical structure, including molecular weight, amylose content, and degree of branching. The amylose content in oat starch ranges from 25.2 to 29.4%, depending on the genotype, environment, and analytical methods used [22]. The digestion rate is associated with the branch chain size of amylopectin, and shorter chains can result in higher digestion rates [23]. Xu et al. [24] investigated the relation between the molecular structure of oat starch, its size and chain length distribution of amylose and amylopectin molecules, and its corresponding digestibility. They found that oat starch contains a high proportion of amylose larger than rice starch amylose, partially contributing to the slower digestion rate. Other factors contributing to the slower digestion of oat starch were fewer short branch chains (DP < 13) and less branching of amylopectin, causing reduced enzyme accessibility. In contrast, a relative lower proportion of the longest branch chains was also observed, which may increase digestibility to some extent. 

Resistant starch (RS) is also able to modulate the blood glucose, and the amount of RS contributes to the GI value of foods. RS accounts for 29.31% of the starch content in raw granular form of oat starch [25]. The RS in raw oat starch is named RS2 starch, where its slow digestion is mainly due to the compact nature of the starch granules making the starch less accessible to enzymes. Since the amylose–lipid complex is resistant to enzymatic breakdown, the high lipid content in oats (3–7%) may be another reason why oat has a relatively high level of RS starch. This type of RS is called RS5 [26]. In addition to being less susceptible to enzymatic breakdown in the upper gastrointestinal tract, the effect of RS on GI is also subject to modulation by gut microbiota, as described in several publications [27,28,29]. Although RS2 occurs naturally, most of the starch needs to be cooked for consumption. In this case, RS3 that is formed due to the recrystallization of gelatinized starch is more commonly consumed by processing via gelatinization and retrogradation, which will be discussed in the following sections.

### 2.2. Oat β-Glucans

β-glucans are found in the cell walls of endosperm and aleurone layers of oats, accounting for 1.73–5.70% of oat grains on the dry basis [30]. They are linear polysaccharide consisting of D-glucose units with β-(1, 3) and β-(1, 4) linkages. β-(1, 4) bonds connect D-glucose monomers to form either cellobiose or cellotriose units, and these units are connected by β-(1, 3) bonds to form β-glucans. The presence of β-(1, 3) bonds eliminates molecular aggregation and makes the molecule water-soluble [31,32]. Differences in the chemical structure of oat β-glucans leads to varied physicochemical behavior. For instance, the ratio of β-(1, 3) and β-(1, 4) linkage gives rise to the water-soluble property and higher molecular weight contributes to increased solution viscosity [33]. 

Among oat ingredients, β-glucans are the most frequently discussed components due to their widely known health benefits, such as anticancer properties, regulation of hypertension, and prevention of cardiovascular disease [34]. The ability of β-glucans to reduce GI have been extensively studied, and numerous researchers have confirmed the effect. Several mechanisms were proposed to explain the effects of β-glucans on GI (Figure 2). The most convincing explanation is that the high viscosity of β-glucans solutions slows gastric emptying, delays the digestion of food matrices, and thus reduces the glucose releasing rate caused by starch hydrolysis. The presence of oat β-glucans increases chyme viscosity and thus retards the rate at which the stomach is emptied at the beginning of the digestive tract. As the digesta continues flowing into the intestine, the high viscosity of β-glucans could then interfere with the transfer of released glucose to enterocytes, resulting in a more steady and less fluctuated glycemic response [35]. From another perspective, as a soluble dietary fiber, oat β-glucans are not digested in the upper gastric tract but instead can be consumed by gut microbiota in the colon. This kind of prebiotic can be fermented by the colonic microbiota, resulting in the production of short chain fatty acids (SCFA) metabolites. Many studies have confirmed that SCFAs such as butyric acid, propionic acid, and acetic acid can regulate the expression of insulin-sensitive glucose transporter type 4 (GLUT-4) which in turn maintains the glucose concentration gradient across cell membranes and enables glucose transport [36]. There is also a piece of evidence that oat β-glucans can activate the PI3K/AKT signaling pathway. Decreased PI3K/Akt activity plays a key role in the pathogenesis of diabetes. The intake of β-glucans could restore decreased PI3K/Akt and thus be beneficial for the management of diabetes through diet [37].

β-glucans as a natural ingredient has gained several official health claims. For example, the Food and Drug Administration (FDA) of the US has approved a health claim that the consumption of at least 0.75 g per portion or 3 g per day of oat and barley β-glucans can reduce the risk of coronary heart disease [38]. The European Foods Security Agency (EFSA) stated that foods including oat and barley β-glucans reduce postprandial blood glucose [39]. In addition to work examining the direct effect of oat β-glucans as food stuffs on the human glycemic response, several studies also investigated their effects when used as a component of various foods. There is much evidence that the inclusion of oat β-glucans can help to reduce the GI value of foods (Table 1). Dose and molecular weight are two significant factors related to the hypoglycemic function of oat β-glucans. The GI value of a food tends to decrease as oat β-glucans dosage increases. More strikingly, increased molecular weight contributes to more viscous food matrices and produces a profound reduction in GI [40]. Despite this effect, the minimum effective dose of oat β-glucans remains a subject of debate. In instant oatmeal, Wolever et al. [41] investigated the dosage effect of oat β-glucans derived from oat bran. They found that each gram of oat β-glucans reduced the incremental area under the blood glucose response curve (iAUC) by 7% and the peak-rise response by 15%. They concluded that 1.6 g oat β-glucan was required to reduce iAUC by ≥20%, whereas only 0.4 g was required for the reduction of 20% in the peak response. A recent meta-analysis including 93 trial comparisons (*n* = 432) indicated that consuming foods containing oat β-glucans reduces glucose iAUC by an average of 23%. Per 30 g available carbohydrate containing oat β-glucans of <1.5 g, 1.5 to <2.5 g, 2.5 to <3.5 g, 4.5 to <5.5 g, and >5.5 g contributed to iAUC reductions of 9%, 14%, 17%, 31%, and 39%, respectively. Low molecular weights (<300 kDa) had no effect, but medium (300 to <1000 kDa) and high molecular weights (>1000 kDa) caused significant reductions of 23% and 32%, respectively [42].

### 2.3. Proteins

Proteins play a significant positive role in controlling blood glucose response. It is widely accepted that dietary proteins can balance blood glucose by slowing the gastric emptying rate, promoting the secretion of insulin, and affecting the digestibility of starch [48]. Dietary protein has insulinotropic effects. The release of several hormones such as glucose-dependent insulinotropic peptides (GIP), glucagon-like peptide 1 (GLP-1), and insulin can be stimulated by dietary proteins [49]. Recently, a claim raised by the American Diabetes Association states that “ingested protein appears to increase insulin response without increasing plasma glucose concentrations” [50]. Moreover, consuming dietary protein increases satiety and slows the gastric emptying rate [51,52]. It is also widely believed that proteins can interact with starch and thus delay the availability of starch to enzymes [53].

Oats are an excellent protein source among cereal crops. High protein content (12–17%) and a more balanced amino acid profile enable oats to be an ideal nutritional ingredient for both animals and humans. The lack of gluten makes them favorable for individuals with celiac disease. Oat proteins can be further fractioned into globulins, prolamins, albumins, and glutelins. Among these fractions, globulins and prolamins take up the largest proportions of 70–80% and represent 4–14% of total proteins present [54]. More recently, oat milk has become an increasingly popular plant protein beverage due to its distinctive taste versus traditional legume-based beverages. Oat milk is preferred by consumers as a plant-based coffee creamer. Although the content of protein in oat milk is lower than that in animal-derived milk, the content of this nutrient in oat milk is close to that in soy milk and could be a viable substitute for animal milk [55]. Although many studies have confirmed the ability of dietary protein to lower glucose blood levels, there are few studies of this effect with oat proteins. However, from the few publications available, evidence suggests that oat proteins are effective. For instance, Tan et al. [56] studied the influence of plant protein on attenuating glycemic and appetitive responses. They found that oat proteins stimulate the release of insulin. A higher protein content in sugar-sweetened beverages enhanced satiety and tended to suppress hunger and desire-to-eat sensations. They further found that the increased insulin was in accordance with the pattern of GIP and GLP-1 release. Thus, the insulinotropic capability of oat amino acid profiles is a fertile field for further research.

### 2.4. Phenolics

Oats are rich sources of phenolics including flavonoids, phenolic acids, lignans, and avenanthramides. These phytochemicals possess high antioxidant capacity, and their content largely depends on plant genotype and production environment [57]. Growing evidence indicates that naturally occurred phenolics have the potential to modulate carbohydrate digestion and glucose absorption through the inhibition of starch digestive enzymes and potential regulation of intestinal glucose transporters [58,59]. More recently, a systematic review and meta-analysis revealed that phenol levels in foods were negatively correlated with GI, and the mechanism was the inhibition of α-amylase and α-glucosidase [13]. Intake of 1 mg/mL of oat phenolics (either caffeic acid, chlorogenic acid, or ferulic acid) significantly suppressed (35–85%) glucose transport in rat intestinal brush boarder membrane vesicles [60]. Bound phenolic compounds exhibited lower susceptibility to enzymes in the human gastrointestinal tract whereas free phenolics were more readily used. Therefore, enhancing the bioavailability of phenolics is crucial to enhance their function. Up to 30% of the total phenolics in oats are free phenolics, and these are more able to raise postprandial plasma phenolic concentration and antioxidant capacity [61]. The content of free and bound phenolics in oats were reported to be 7.4−197 and 377−2320 μg/g, respectively. Avenanthramides and ferulic acid were the major oat phenolics found in both bound and free form. Free and bound phenolics extracted from oats inhibited the in vitro maltose hydrolysis using digestive enzymes in rat intestinal powder and attenuate glucose transport [62]. Pearling lowered the avenanthramides content compared with whole oat grains [63]. In addition, free and bound phenolic acid from naked oat inhibited the activity of α-amylase and α-glucosidase and therefore decreased starch digestibility [64].

## 3. Processing of Oats and Effects on GI

**Figure 3:**
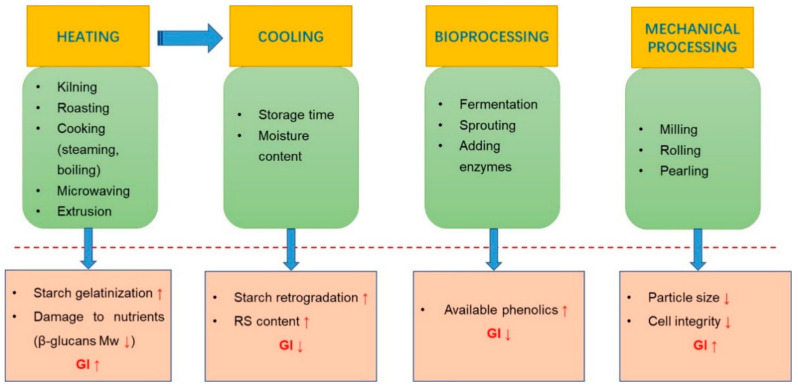
The overview of the effects of representative processing techniques on GI and possible mechanisms.

To meet consumer requirements, extend the shelf life, and increase product diversity, a variety of processing techniques have been applied in oat products. Different levels of processing may cause a change of nutritional composition and physical structure, thus greatly affecting the glycemic response in humans (Figure 3) [65]. Processes commonly applied in oats include preparatory treatments (e.g., milling and rolling), heat treatment using raw oats, and biological treatment (e.g., fermentation) [66,67]. Preparatory treatment is used mainly to physically change the structural format of foods to produce different particle sizes or disrupt cell integrity. These techniques are totally associated with the structural form of foods, and their corresponding impact on GI is discussed in the next section in the present review. Therefore, this section will consider heating, cooling, and bioprocessing on GI.

### 3.1. Heating

One purpose of heat treatment of oats is to inactivate endogenous enzymes, especially lipase, to prevent rancidity and extend shelf life. Another reason for such treatment is to produce products that consumers find more convenient, such as those that are easy to prepare [68,69]. Under the heat treatment, starch is gelatinized to some extent. The starch granules swell as water is absorbed, amylose molecules leach out, and the starch granules disintegrate. Accordingly, heat treatment improves the availability of starch for enzyme hydrolysis and therefore increase GI compared to raw products. In addition, harsh heat treatment conditions can destroy the structural integrity of nutritional components and reduce their functionality. The degree of this effect is greatly influenced by temperature, moisture, and duration period.

Kiln drying is a typical step in oat processing. It inhibits lipase activity and prevents rancidity and corresponding off-flavor by imposing a certain temperature and moisture on raw oats. Such treatment can also produce a pleasant caramel aroma. The consistency of flavor and nutritional quality of the final oat products is largely dependent on the proper control of temperature, moisture content, and treatment period. Kiln drying is done typically at about 100 °C for up to about 2 h [1]. Kilning at 106 °C in a convection oven for 120 min reduced large starch granule content and the molecular weight of amylose, generating a product with an estimated GI of 85.55 [70]. In addition, Nguyen et al. [71] investigated the influence of heat treatment on oat starch structure by steaming groats at 100 °C for 40 min with moisture of 18–19%, dry heating at 125 °C for 10 min, and finally drying at 112 °C for 30 min to a moisture content of 11%. They found that this treatment generated shorter amylopectin chains, higher amounts of long amylose chains, and higher amylose content. Traditional cooking methods such as boiling, steaming, and autoclaving require heat and hydration. This hydrothermal treatment causes starch to gelatinize more easily. Ovando-Martínez et al. [70] found that the GI values of oats steamed at 106 °C and autoclaved at 120 °C were 80.9 and 100.4, respectively. Autoclave treatment increased the transition temperature and digestibility of oat starch. Unlike traditional convectional heating, microwave heating produces superheated micro-sites that facilitate energy transfer. Harasym and Olędzki [72] compared the glycemic response of conventional and microwave-heated oatmeal and found that oatmeal heated for 3 min in a microwave exhibited a lower GI of 62.2. 

Another treatment commonly applied in oats is extrusion. Extrusion cooking with high heat, pressure, and shear effects enhances starch gelatinization and structural changes, and this technique is used to make ready-to-eat oat products. Extrusion at 18% moisture content, 155 °C barrel temperature, and 150 rpm screw rotating speed gave the gelatinization degree of 96.92% in oat flour and led to a much easier release of glucose [73]. However, harsh processing conditions such as extrusion can depolymerize β-glucans by breaking glycosidic bonds [74]. This depolymerization reduces the molecular weight of β-glucans with a corresponding decreased in apparent viscosity. Brummer et al. [74] observed an inverse relationship between the glycemic response and molecular weight of β-glucans.

During thermal processing, the GI of oat products is tightly associated with the degree of starch gelatinization that in turn affects the level of glucose releasing. Of these heat treatments that are commonly applied in oats, dry-heat treatment such as kiln drying may cause a more moderate impact on GI than hydrothermal treatment, including boiling and steaming, since the higher moisture facilitates the starch gelatinization and consequent digestion rate. Microwave heating is more likely to produce a lower GI than convectional heating because the structural change of starch seems different under microwave heating, but more studies are required on how this technique affects GI. Extrusion treatment produces a higher GI under the action of heat, shear, and pressure, due to the high gelatinization of starch and the depolymerization of β-glucans. Overall, reducing the starch gelatinization and corresponding digestibility by controlling the heat temperature, moisture, and time is an effective way to produce low-GI foods, which is beneficial to the management or the prevention of Type 2 diabetes.

### 3.2. Cooling

The cooling process is conducted generally after heating. In food processing, this step removes the thermal source by either naturally cooling or using a cooling apparatus. For oat products, cooling is commonly used for highly gelatinized foods with medium or high moisture content, whereas it is not suitable for slightly gelatinized products such as oat flakes. For instance, cooling is often used in the production of extruded oat noodles for the Chinese market to improve the texture and mouthfeel of the final product. Due to the lack of glutens, extruded oat noodles are formed by using the network structure generated from starch gelatinization. The starch retrogradation achieved by cooling makes the noodle structure more compact. During this process, the structure of gelatinized starch in food is rearranged to form a more structured matrix. This retrogradation of starch not only changes the texture but also affects digestibility and glycemic response. Cooling is a crucial determinant of starch retrogradation. At lower temperatures, the gelatinized starch is more easily to rearrange under the action of water molecules to form a relatively ordered structure. The degree of starch recrystallization increases with storage time. At a certain temperature, the retrogradation of amylose occurs mainly within a short storage period, while that of amylopectin happened in long-term storage. Therefore, the reduced digestibility is mainly the result of short storage. The retrogradation rate is also dominated by water content. Retrogradation is reduced by both low water content due to the restricted movement of starch molecules and high water content due to a dilution effect. Cooling is a potential strategy for producing low-GI foods, because this treatment allows the formation of starch retrogradation and consequently less digestibility. The retrograded starch is often less digestible, since a more ordered starch structure has decreased susceptibility to digestive enzymes. The most common cooling temperatures are 4 °C, 25 °C, and 30 °C; of these, 4 °C can produce a faster retrogradation rate of amylopectin [75]. This process is inhibited when the temperature is below freezing, as starch mobility is reduced within solid water molecules, and thus, their rearrangement is difficult. Compared with other common cereals, oat starch requires the lowest temperature (55 °C) to reach 50% gelatinization. Even so, the degree of oat starch retrogradation is limited compared to that of other cereal grains, because the presence of lipid interferes with the process to a certain extent [76]. The degree of retrogradation in oat starch was substantially lower than that in wheat starch (42.04%) when stored at 4 °C [77]. The retrogradation of amylose can generate type 3 resistant starch (RS3). It was reported that RS3 showed the therapeutic effect on Type 2 diabetes through improving dyslipidemia, reducing insulin resistance, and increasing insulin sensitivity [78]. Compared with other plants, especially legumes, the RS3 content formed in oats is relatively low [79]. Processing conditions, i.e., heat treatment and the corresponding cooling process, play a paramount role in the formation of RS3. Dual autoclaving–retrogradation treatment can increase the RS3 in oats from 25.81% to 38.88% [80].

### 3.3. Bioprocesssing

Bioprocessing, including germination and fermentation, can effectively enhance the nutritional value and functional characteristics of oats. This kind of processing uses bioactive substances such as endogenous enzymes and microorganisms to change the nutritional composition and create a variety of functions [81].

Fermentation is a metabolic process that brings about a desirable change to a foodstuff under the activity of microorganisms. Yeast and lactic acid bacteria are commonly used to fermented oat products. There are few reports on the effect of fermentation on glycemic response in oats. The fermentation of oat flakes with *L. plantarum* LP09 increased polyphenol availability and the antioxidant activity and decreased the hydrolysis index, showing a potential for reduced GI [82]. The organic acids generated from fermentation showed potential to reduce GI by reducing the degree of starch gelatinization, increasing the formation of RS, and inhibiting digestive enzymes by the increasing phenolics [83]. The phenolic content in oats was increased by 59.1% after 12 days of solid-state fermentation with Monascus [84]. 

Germination is the process of growth from a plant seed to seeding. This process breaks down grain components into smaller substances through the action of endogenous grain enzymes when water, oxygen, and favorable temperatures are present. Germination alters nutrient availability, for example through the degradation of polysaccharides, and it decreases antinutrients [81]. These factors collectively determine the blood glucose response of the final product and, therefore, the effects of germination on GI are multifaced. It is widely accepted that germination enhances the bioavailability of phenolics, which in turn increase inhibitory effects on amylase. Germination produces multiple phenolics that exhibit potent inhibition on a-amylase and a-glucosidase [85]. During oat germination, bound phenolic decrease and free and total phenolics increase, especially p-coumaric, ferulic acids, and avenanthramides [84]. However, excessive germination can lead to the depolymerization of β-glucans due to the action of endogenous β-glucanase [83], which would adversely affect the GI of a product. Therefore, properly controlling the degree of germination by adjusting time, temperature, and moisture is a potential strategy to optimize oat foods for low GI.

In addition to natural biological processes, the direct addition of external enzymes is frequently used in the processing of oat products. This process is more commonly used for oat milk production. External enzymes such as α-amylase can help generate sweet flavors by catalyzing the partial hydrolysis of starch. Treatment with α-amylase improved the content of extractable phenolic compounds and the corresponding antioxidant capacity of oat flour [86]. Carbohydrate-hydrolyzing enzymes can decompose the cell wall structure and facilitated the release of phenolics [87]. The corresponding inhibition effect on digestive enzymes could further reduce GI values. However, free monosaccharides and oligosaccharides produced by enzymatic hydrolysis may have the opposite effect.

These bioprocessing techniques are drawing more and more interest to oat products, since they can change the quality and quantity of nutritional constituents. With regard to their effects on GI, current evidence mainly attributes them to oat phenolics. These bioactive components can reduce the absorption of glucose by inhibiting digestive enzymes, which is a potential therapeutic way to manage Type 2 diabetes [13]. However, more clinical trials are still needed to confirm the efficiency of this processing technique on oat GI.

## 4. GI Values of Different Oat-Based Foods

In the United States, oats emerged as a food for the table in the 19th century. By virtue of its low cost and good flavor, oat-based breakfast cereals have gradually become a favorite food. With improved public awareness of the health benefits of eating oats, the oat processing industry has thrived. Not only does the traditional oatmeal and oat beverage market continue to expand, but new oat products, such as oat bars and oat rice, are also emerging. Based on the format of the ingredients, oat-based foods can be mainly classified as grains, flakes, flours, and other types such as oat bran. The production of these different ingredient formats mainly involves physical mechanical processing, such as pearling, rolling, and milling. A variety of oat products can be derived from these processed ingredients, and the corresponding size and thickness of the ingredient units determine to a large extent the GI level of the final product. The structural shape is closely associated with particle size in food matrices, and a more fragmentized structure increases the surface area and exposure of starch granules to amylolytic enzymes during digestion, leading to higher GI values. In this section, we discuss the GI of various oat-based foods by considering how it relates to the food format.

### 4.1. Oat Grains

After harvesting, dehulling, and cleaning, oat grains are ready either for consumption or further processing. As the least processed type, the intact grain structure maintains its original shape and retains the nutrients to the greatest extent. However, whole oat grains have a thick aleurone layer that slows water absorption and starch gelatinization during cooking. Whole grains also have a relatively hard texture and poor mouthfeel, and thus, the intact grain has not become a mainstream consumer product. Despite these challenges, a few whole grain-based oat products have emerged and captured certain markets by employing appropriate technology. For instance, pearled or “oat rice’’ has gained growing popularity in China with an annual consumption of 200,000 tons, since this product is compatible with the eating habits of Chinese consumers. Pearled oats is a whole-grain product processed by cleaning, dehulling, polishing, and inactivation of enzymes. Polishing is the most critical process as it removes the fluff (plant trichomes) and a specific amount of bran from the surface of oat grains, enabling the grain to absorb water and cook more quickly. This kind of product can be cooked with rice to produce a food with a special oat flavor and reduced GI value [88]. Pearled oats also must undergo an enzyme deactivation process, such as steaming, because the absence of the bran layer promotes the oxidization of lipids in the grain, which generates rancidity [89,90].

In general, intact oat grain products have relatively lower GI values ranging from 43.4 to 64.6 (Table 2). Zhu et al. [91] compared the glycemic response of whole grain oats and pearled oats using different cooking conditions. They found that the GI values of both products cooked under either high (70 kPa) or normal pressure were less than 58. No significant difference was found even after either bran removal by polishing or high-pressure cooking, suggesting that whole grain oats and pearled oats could be used in glycemic management diets. In addition to clinical human GI tests, the in vitro estimated glycemic index (eGI) test was also used to evaluate grain-based oat products. Although the accuracy of the in vitro test has yet to be fully validated, it can offer an approximation of in vivo results by taking advantage of advanced bionic models. Our previous research applied the Dynamic In vitro Rat Stomach–Duodenum (DIVRSD) model to assess the GI value of oat-based foods by mimicking the physical movement and physiological conditions found in vivo to simulate in vitro digestion. The eGI of whole oat grains was 43.4, whereas that of pearled oats was 64.6 [92]. Current evidence suggests that oat grain products are low or medium GI foods for the following reasons:

(1) The grain structure is compact and intact, and the food can only be chewed and ground through the mouth. This minimal process ensures an intact cell wall and maintains a barrier to starch, thus reducing the accessibility to digestive enzymes and producing a lower GI value [64],
(2) The outer aleurone layer of oat grains acts as a barrier to water absorption and starch gelatinization, thus retarding the starch hydrolysis and the release of glucose, and
(3) The β-glucans and polyphenols-rich bran layer can delay gastric emptying and inhibit the activity of digestive enzymes [93].

### 4.2. Oat Flakes

Oat flakes, also called rolled oats, are the most common oat product and are widely accepted by consumers worldwide. Oat flakes are made from whole oat grains (larger size) or steel-cut oats (smaller size) by being rolled into flat flakes with thicknesses ranging from 0.4 mm to 1 mm [88]. Steel-cut oats are produced by being chopped with steel blades into small, pinhead-sized pieces from whole oat grains, which can be directly used either to make porridge or further rolled into smaller flakes. Here, we classify steel-cut oats in the category of oat flakes. Rolled oats can be categorized either as “old-fashioned”, “quick”, or “instant” based on the cooking time required. In general, old-fashioned oats denote lightly processed rolled whole oats that can be directly cooked into a porridge. Highly processed rolled oats have a shorter cooking period and are called quick or instant oats. Before being flaked, several steps including cleaning, dehulling, and kilning are required to produce oat flakes. As a result of the high content of lipids in oats, the kilning process is necessary to denature endogenous enzymes and extend shelf life. Kilning also softens the texture of the flakes, thus enhancing mouthfeel and partially gelatinizing the starch in the product.

Compared with oat grains, flake-shaped products undergo higher levels of processing and bear greater loss of nutritional properties. Heat treatments such as kilning or steaming can degrade some measures of nutritional quality. For example, flakes tend to exhibit higher GI values versus whole oat grains with a wide range from 44.7 to 114 (Table 2), largely depending on the cooking conditions and the flake thickness. Thickness is a crucial determinant of the GI value. According to the study of Granfeldt et al. [94], the GI values of thicker flakes (1 mm, GI = 72–78) were significantly lower than that of thinner flakers (0.5 mm, GI = 99–114). Thinner flakes and smaller particle size allow oats to hydrate more quickly, thus accelerating starch gelatinization. In addition, in thicker oat flakes, the outer layer of the endosperm and/or the cell walls are less disrupted, thus protecting the starch granules from enzymatic hydrolysis. Cooking conditions are another key factor affecting the GI value. As noted previously, pre-heat treatment such as kilning enables the partial gelatinization of starch. The pre-cooking that is applied to quick or instant flakes results in higher GI values for these produces compared to raw flakes [12]. Harasym and Olędzki [72] compared the effect of conventional heating and microwave heating on the glycemic response of oat flakes. They found that flakes heated for 3 min in a microwave had lower GI than those heated for 5 min in a microwave and those heated for 7 min by convection. Ultra-processing such as extrusion may cause a maximum degradation of GI resulting in values as high as 105 [95]. Steel-cut oats show lower GI values than flakes probably because the lightly broken structure and the lack of pre-heat treatment help maintain cell wall integrity.

### 4.3. Oat Flours

Whole oat flours are produced with stone or hammer milling followed by mesh screening. The ground flours can serve as ingredients for other more processed products. This kind of ingredient is popular in Asian countries, including India and China. In India, whole grain oat flours are used to produce oat bread know as ‘jarobra’. In China, oat flours are commonly used to produce traditional foods, such as oat noodles and “oat wowo”, which is a steamed thin sheet of oat dough. Oat wowo and oat noodles are mainly consumed as staple foods in northern China, especially Inner Mongolia. Flour-based foods account for 70% of total oat foods with an annual production of 0.22 million tons in the Chinese market [96]. Oat flours are made by cleaning, dehulling, tempering, roasting, and then grinding and sieving. The processing of traditional Chinese oat flour-based foods includes three thermal treatments: roasting the oat grains, mixing the dough with hot water, and either boiling or steaming the final product. Roasting oat grains inactivates endogenous lipase, reduces clogging of the pulverizer screen, improves the flour yield, and extends the shelf life. Since oats lack glutens, the flours cannot form dough such as wheat flour. Therefore, mixing the dough with hot water enables the starch to partially gelatinize and form a dough network. Finally, boiling or steaming makes the starch fully gelatinized and ready for consumption [97].

Currently, the GI values for only a few oat flour products are known, which are probably due to the small consumer base for such products. From the limited data generated in both in vitro and human trials, GI values range from 53.6 to 92. Oat mueslis or muffins show lower GI values, which is likely due partially to their supplementation with other ingredients such as raisins and dried fruits. Although the Dynamic In vitro Rat Stomach–Duodenum (DIVRSD) model gave relatively lower values of 54.4 and 61.8 [92], other human clinical trials showed a far higher value of 74 [98]. Moreover, unpublished studies in our laboratory revealed that whole oat flour extruded noodles have an even higher GI of 86.6. Therefore, more tests are needed to resolve these differences by more comprehensively assessing the GI of oat flour products. It is generally believed that milling reduces particle size and produces a larger surface area, thus enhancing access to digestive enzymes and inducing an increased glycemic response [99]. The release of glucose increased as particle size decreased from 2.8 to 0.125 mm [100]. Meanwhile, the smaller particles allowed greater hydration and more thorough gelatinization.

### 4.4. Oat Bran

Oat bran is produced from grinding oat grain and separating the outermost bran layer from the rest of the grain. It can also be made from rolled oats. The corrugated rolls are reduced to the particle size of the steel-cut groats, and the coarse fraction, consisting of the oat bran, can then be separated [88]. Oat bran became commercially available in the 1980s. It has a total β-glucan content of at least 5.5% and a total dietary fiber content of at least 16% [101]. This abundant β-glucans and dietary fiber content made oat bran enormously popular. In addition to being consumed as a commercial hot cereal product, oat bran is frequently incorporated into bread, cookies, and other snack foods.

In human clinical trials, the GI values of oat bran ranged from 34 to 66.3, which is lower than most other oat food matrices. In a clinical GI test using 12 hyperlipidemic patients, the value of oat bran was 59. Consuming commercial oat bran containing 22% β-glucans before meals in amounts of 4.5, 13.6, or 27.3 g remarkably reduced glycemia. When converted to β-glucans content in bran, each gram of β-glucan reduced the blood glucose iAUC by 4.35% [67,102]. In addition, the supplementation of deep-fried dough/batter food with up to 20% oat bran significantly reduced the amount of rapidly available glucose in vitro and increased the amount of slowly available and unavailable glucose. Fried dough containing oat bran had an eGI of 56.34–57.27, which was lower than that of the control sample [103]. It is commonly accepted that selecting oat bran as a food alternative may be useful for the management of hyperglycemia. Enrichment with dietary fibers, particularly oat β-glucans, is the major reason why oat bran supplementation can reduce the GI value of foods [72]. This nutritional feature is responsible for the high viscosity of oat bran, which in turn reduces the gastric emptying rate. As a result, oat bran supplementation in foods is an effective strategy for increasing oat β-glucan content. As with β-glucans in general, the quality and quantity of oat β-glucans in oat bran determines how glycemic behavior is affected. Therefore, processes such as extrusion may affect the molecular weight of the β-glucans and thus change the GI of oat bran [74]. In addition, the bran layer is also a primary source of oat phenolics, and these compounds may reduce the GI by their inhibitory activity against α-amylase and α-glucosidase [104].

**Table 2:** GI values of oat-based products with different structural formats.

Format	Food Matrices	Key Cooking Conditions	Available Carbohydrate/g	Subjects/In Vitro Model ^1^	GI/eGI	Ref.
Grain	Whole grain oats	Steaming for 50 min	50	10 N	51	Zhu et al. [91]
	Whole grain oats	Steaming under 70 kPa of pressure for 20 min	50	10 N	52	
	Pearled oats	Steaming for 50 min	50	10 N	51	
	Pearled oats	Steaming under 70 kPa of pressure for 20 min	50	10 N	58	
	Whole grain oats	Roasting at 160 °C for 15 min		In vitro dynamic rat stomach–duodenum model	43.4	Feng et al. [92]
	Pearled oats	Cooking for 30 min		In vitro dynamic rat stomach–duodenum model	64.6	
	Pearled oats		50	10 N	51.5	Zhang et al. [105]
Flake	Porridge, made from steel-cut oats		33	9 N	52	University of Sydney [106]
	Steel-cut oats		23	30 N	53	Wolever et al. [107]
	Old-fashioned oats		23	30 N	56	
	Instant oats		23	30 N	67	
	Porridge made from rolled oats	Cooking for 20 min	23	6 N	49	Jenkins et al. [5]
	Oatmeal batch bread		25	10 N	62	Henry et al. [108]
	Oatmeal (0.5 mm)	Steaming for 30 min and baking at 140 °C		In vitro dynamic rat stomach–duodenum model	44.7	Feng et al. [92]
	Oatmeal (unpackaged)		50	9 N	55	Yang et al. [109]
	Oatmeal		50	8 N	83	
	Rolled oats		19	10 U	59	University of Sydney [106]
	Oat flakes	Convection heating for 7 min	25	12 N	75.7	Harasym and Olędzki [72]
	Oat flakes	Microwave for 3 min	25	12 N	62.2	
	Oat flakes	Microwave for 5 min	25	12 N	75.1	
	Oatmeal porridge (0.5–0.6 mm)		50	12 N	74	Hätönen et al. [110]
	Oat flakes (0.5 mm)	Roasting and steaming	50	10 N	114	Granfeldt et al. [94]
	Oat flakes (0.5 mm)	Soaking and roasting	50	10 N	99	
	Oat flakes (1 mm)	Soaking	50	10 N	78	
	Oat flakes (1 mm)	Soaking and roasting	50	10 N	72	
	Oat flakes (1 mm)	Steaming	50	10 N	76	
	Extruded oat flakes	Heating at 83 °C followed by extrusion	25	In vitro adult fasted dynamic gastric model	105	Ballance et al. [95]
Flour	Oat wowo	Roasting at 160 °C for 15 min and steaming for 10 min		In vitro dynamic rat stomach–duodenum model	54.4	Feng et al. [92]
	Oat paste	Extrusion at 160 °C		In vitro dynamic rat stomach–duodenum model	61.8	
	Oat bread	Baking for 45 min at 190 °C		In vitro starch digestibility	71	Wolter et al. [80]
	Oat muesli		50	19 N	55	Tan et al. [111]
	Muffin	Baking for 20 min at 180 °C	50	12 N	53.6	Soong et al. [112]
	Oat flour	Heating at 83 °C	25	In vitro adult fasted dynamic gastric model	92	Ballance et al. [95]
	Wholemeal oat flour porridge	Boiling for 2.5 min	30	8 N	74	Liljeberg et al. [98]
Other types	Oat bran	Convection heating for 7 min	25	12 N	61.9	Harasym and Olędzki [72]
	Oat bran	Microwave for 3 min	25	12 N	49.1	
	Oat bran	Microwave for 5 min	25	12 N	66.3	
	Oat bran + milk	Extrusion (temperature = 181 °C, water = 18.7%, mechanical energy = 135 Wh/kg; molecular weight of β-glucans = 2180 kDa)	31	12 N	34	Brummer et al. [74]
	Oat bran + milk	Extrusion (temperature = 220 °C, water = 14.5%, mechanical energy = 125 Wh/kg; molecular weight of β-glucans = 921 kDa)	31	12 N	43	
	Oat bran + milk	Extrusion (temperature = 228 °C, water = 10%, mechanical energy = 145 Wh/kg; molecular weight of β-glucans = 627 kDa)	31	12 N	45	
	Oat bran + milk	Extrusion (temperature = 237 °C, water = 7%, mechanical energy = 148 Wh/kg; molecular weight of β-glucans = 326 kDa)	31	12 N	44	

Note: ^1^ N = normal subjects, U = unknown, D = diabetes subjects.

## 5. Conclusions and Future Research Need

In summary, the components of oats contribute to the GI of a final food product to varying degrees. These components can interfere with starch hydrolysis and the corresponding absorbance of released glucose and increase product viscosity, causing a reduced gastric emptying rate. We evaluated the literature on several typical oat components and their impact on GI. As a critical substance for raising blood glucose, oat starch has a smaller granule size, making it more susceptible to digestive enzymes. Multi-faced factors including crystal characteristics, amylose content, and degree of branching collectively determine the overall GI level. The presence of RS2 and RS5 in raw oats may also contribute to the reduced GI to some extent. Among non-starch components, β-glucans are the most promising substance and have been successfully incorporated into a series of foods to produce low GI products. The β-glucan dose and molecular weight are crucial determinants affecting the viscosity and gastric emptying rate. The higher content of protein in oats is also an important factor that deserves attention. Although there is a lack of sufficient evidence to directly show the effect of oat proteins on glycemic response, it is thought that they can promote the secretion of insulin and increase the viscosity of food to slow gastric emptying. Oat phenolics are also important, and their inhibitory effects on digestive enzymes is possibly the mechanism through which these compounds reduce GI level.

From the standpoint of a manufacturer, processing techniques that range from thermal to biotechnological affect starch digestibility and the activity of functional ingredients, thus modulating the glycemic response. Heat processing facilitates starch gelatinization and increases the GI to varied degrees, depending on the thermal conditions. Harsh conditions such as extrusion can cause the decomposition of other active compounds, such as β-glucans, thus increasing GI values. Cooling is commonly employed in highly gelatinized oat products. This step promotes starch retrogradation and can generate a certain amount of RS3, which reduces the GI level. Bioprocessing, including germination and fermentation, can facilitate the release of phenolics through enzymatic catalysis and thus exaggerate the inhibitory effect of phenolics on digestive enzymes.

From field to table, oats are processed into various foods for consumption, and these foods exhibit high variability of GI values. The various physical formats of oat products are from the result of different degrees of physical and mechanical alteration. Grain, flake, and flour are typical ingredient forms for oat products. Minimum mechanical processing (grain) maintains the intact cell wall and larger particle size that reduce the accessibility to enzymes and thereby tend to enable lower GI values. In contrast, ultra-processing makes the starch more easily hydrated and increases the surface area available to enzymes. Another typical product, oat bran, affects GI behavior through the enrichment of dietary fibers. The systematic summary of GI of oat products with different physical formats presented in this review helps to synthesize knowledge of the field and provides practical guidance useful for management of diabetes with diet.

The prevalence of diabetes has driven efforts to develop functional foods with the potential to lower glycemic response. For decades, oats have been used in efforts to reduce the GI of popular foods. Attempts to unravel the GI of oat-based foods have considered both the components of oats and the processing techniques used to generate food products. Tremendous research effort has been placed on β-glucans. The starch parameters and other bioingredients are also core factors affecting glycemic modulation, and their individual contributions are gradually becoming clearer. The lack of extensive knowledge of the interaction and co-contributions of the multiple components in oats are a challenge to our complete understanding of the GI effects of this important food ingredient. Future work should assess the multiple contributions of the various functional ingredients within oats and account for their synergistic or antagonistic effects on the GI of final products.

## Figures and Tables

**Table 1:** Inclusion of oat β-glucans in varied food matrices and corresponding GI values.

Food Matrix	Content per Available Carbohydrate/%	Molecular Weight/kDa	GI/eGI	Subjects/In Vitro Model ^1^	Key Findings	Ref.
Yeast leavened bread	2.5	282	64	13 N	The addition of oat β-glucans decreased GI by 32–37% compared to white bread.	Ekström et al. [43]
	3.5		68			
	4.5		63			
β-glucan-enriched breakfast cereal and bar	7.3		52	16 D	The GI of the test foods used in this study decreased by 4.0 ± 0.2 units per gram of β-glucans, compared with commercial oat bran breakfast cereal.	Jenkins et al. [44]
	6.2		43			
Oat flour	4	890	85.7	In vitro starch digestibility	β-glucans slowed the rate of starch digestion.	Kim and White [45]
	5.3	980	82.7			
	7.4	1150	77.2			
	7.7	770	78.3			
Oat bran products	1		83.7	10 N	The glycemic responses to oat products with increasing amounts of β-glucans had lower peak values than the reference glucose load.	Mäkeläinen et al. [46]
	2		58.3			
	3		63.6			
Snack bar	0		75	12 N	Incorporation of 1.5 to 6 g of β-glucans into snack bars had no additional glucose-lowering benefits irrespective of dose and source compared to the control bars (0 g β-glucans).	Panahi [47]
	0.75		77			
	1.5		80			
	3		71			
Oat starch	20	113	85.6	In vitro starch digestibility	Viscosity attributed to the β-glucans Mw reduced starch hydrolysis during in vitro digestion.	Kim and White [40]
		698	82.8			
		904	68.4			

Note: ^1^ N = normal subjects, U = unknown, D = diabetes subjects.
